# The “virtual lesion” approach to transcranial magnetic stimulation: studying the brain–behavioral relationships in experimental pain

**DOI:** 10.1097/PR9.0000000000000760

**Published:** 2019-08-07

**Authors:** Irit Weissman-Fogel, Yelena Granovsky

**Affiliations:** a Physical Therapy Department, Faculty of Social Welfare and Health Sciences, University of Haifa, Haifa, Israel; b The Laboratory of Clinical Neurophysiology, Technion Faculty of Medicine, Haifa, Israel; c Department of Neurology, Rambam Health Care Campus, Haifa, Israel

**Keywords:** Transcranial magnetic stimulation, Pain measurements, Experimental pain, “Virtual lesion”

## Abstract

Transcranial magnetic stimulation (TMS) can be used to create a temporary “virtual lesion” (VL) of a target cortical area, disrupting its function and associated behavior. Transcranial magnetic stimulation can therefore test the functional role of specific brain areas. This scoping review aims at investigating the current literature of the “online” TMS-evoked VL approach to studying brain–behavioral relationships during experimental pain in healthy subjects. Ovid-Medline, Embase, and Web of Science electronic databases were searched. Included studies tested different TMS-based VLs of various pain brain areas during continuous experimental pain or when time-locked to a noxious stimulus. Outcome measures assessed different pain measurements. Initial screening resulted in a total of 403 studies, of which 17 studies were included in the review. The VLs were directed to the prefrontal, primary and secondary somatosensory, primary motor, and parietal cortices through single/double/triple/sequence of five-TMS pulses or through repeated TMS during mechanical, electrical contact, radiant heat, or capsaicin-evoked noxious stimulation. Despite a wide variability among the VL protocols, outcome measures, and study designs, a behavioral VL effect (decrease or increase in pain responses) was achieved in the majority of the studies. However, such findings on the relationships between the modified brain activity and the manifested pain characteristics were often mixed. To conclude, TMS–elicited VLs during experimental pain empower our understanding of brain–behavior relationships at specific time points during pain processing. The mixed findings of these relationships call for an obligatory standard of all pain-related TMS protocols for clearly determining the magnitude and direction of TMS-induced behavioral effects.

## 1. Introduction

Transcranial magnetic stimulation (TMS) provides a unique methodological approach for determining the true functional role of certain brain areas, through determining the relationships between brain areas and specific behaviors. Applied as time-locked single pulses or trains of repetitive magnetic stimuli (rTMS) at certain stimulation parameters, TMS can be used to transiently disrupt the function of a target cortical area and consequently its associated behavior. This creates a temporary “virtual lesion” (VL) in an otherwise healthy brain.^55^ Consequently, if a cortical process is contributing to a behavior, TMS of the cortical areas involved will result in a deteriorated performance of that behavior. The first time this type of TMS was applied was in studies of the visual cortex where a functional disruption of the occipital cortex (OCC) blocked the detection of visual stimuli.^1^ Today, this VL approach is generalized to other neurocognitive processes. These include working memory, learning processes, speech and language,^49^ and various sensory modalities such as auditory and somatosensory. Transcranial magnetic stimulation can therefore be considered an excellent noninvasive exploratory tool for testing the functional relevance of specific cortical areas.^55,56^

Transcranial magnetic stimulation is limited by the fact that neither the neuronal structures activated, through inducing a local electrical field, nor the direction of the neuronal modulation (excitation or inhibition) is known with high accuracy. Accordingly, the effects of TMS on a given brain region and a given task may decline, improve, or continue unchanged, depending on whether or not neurons involved in the task become inhibited, excited, or remain unaffected. Based on the several basic reviews that discuss the role of VL in cognitive neuroscience,^70,71,78^ the main factors that can influence the direction of behavioral and neurophysiological effects are: (1) The physical properties of TMS. Based on the results from several animal studies, low-intensity stimulation may result in early excitation followed by long-lasting neuronal inhibition; the opposite is observed in response to high-intensity stimulation. For rTMS protocols, a stimulation frequency at or below 1 Hz results in neuronal inhibition, whereas stimulation at higher frequencies will result in neuronal facilitation. Therefore, the direction of behavioral changes depends on the stimulation properties, the task being accessed, and the functional relevance of the target area. (2) The baseline state of the stimulated brain. When TMS is applied before or at the onset of a behavioral task, all neuronal populations are at their baseline level of activity. This may suggest that all neuronal populations are stimulated, resulting in an increased cortical excitability to the upcoming stimulation; the factors such as fiber orientation, size of axons and neurons, and different type of neurons may influence the extent of neuronal excitation. By contrast, when TMS is applied during task performance, not all the neurons are involved in that process. Because the TMS facilitates preferentially the subliminally activated or relatively inactive neurons (those that are not involved in task performance and are most excitable for the upcoming stimulation, more than if they are already discharged), this activity imbalance adds noise to neural processing, which in turn reduces the signal-to-noise ratio of the given task and consequently disrupts its performance. Therefore, the time point of the TMS stimulation may determine whether the behavioral effect will be inhibitory or facilitatory. (3) The short- vs long-distance neural effects. Transcranial magnetic stimulation may affect adjacent brain areas when the induced neuronal electrical activity is spread locally, or TMS may affect more distant brain areas by the propagation of action potentials along corticocortical and corticosubcortical axons. Therefore, the behavioral effect resulting from a VL of a primarily stimulated brain site may be modified by distant neuronal activity. Whether these long-distance effects are excitatory or inhibitory will depend on how TMS affects the target site.

Generally, in certain rTMS protocols, modulation of the excitability level of a given cortical area lasts well beyond the duration of the rTMS train. These protocols therefore allow us to study the association between brain activity and behavior without applying the intervention during the task. In such protocols, the behavior is evaluated after the rTMS (offline mode). Despite the fact that some researchers refer to this stimulation design as a VL, this review only considers the online mode of VL, defined as a TMS-related disruption of an ongoing behavioral task.

In this study, we reviewed studies that focus on the effect of VL applied during (ie, online mode) experimental pain in healthy subjects. This decision was based on the belief that we first need to understand the VL effects on the brains of healthy individuals before exploring its effects, if tested, on the brains of patients with pain. The studies included were not restricted in terms of their tested outcome measures and therefore, various aspects of central pain processes including intensity coding, unpleasantness, and localization were evaluated in response to VL. Beyond these psychophysical effects, we concentrated on the methodological aspects of the TMS-evoked VL directed to various cortical areas involved in pain processes. We discuss the scientific significance of these VL protocols in the study of pain.

## 2. Methods

### 2.1. Search strategy

An online search of relevant electronic databases including Ovid-Medline (since 1946), Embase (since 1974), and Web of Science (since 1965) was performed. Text words contained in the title and abstract, and of the index terms used to describe articles were searched across all the databases. First, the following keywords and index terms were used: “virtual lesion,” “VL,” “transcranial magnetic stimulation,” “TMS,” “repetitive transcranial magnetic stimulation,” “rTMS,” “magnetics,” “magnetic stimulation,” “cortical stimulation,” and “transcranial magnetic.” This was followed by another search including the terms “pain measurement,” “pain measurements,” “sensory thresholds,” “pain threshold,” “threshold, pain,” “signal detection, psychological,” “nociception threshold,” “nociceptive threshold,” “pain tolerance,” “experimental pain,” “experimentally induced pain,” “pain intensity,” “pain localization,” “pain discrimination,” “laser stimulation,” “pain assessment,” and “pain evaluation.” The final search encompassed the terms “pain,” “acute pain,” “chronic pain,” and “clinical pain.” All articles searched were restricted to the English language. We searched for all keywords separately within each search. Then, we used the logical operator OR between all the keywords in each search. Thereafter, the logical operator AND was used to combine the results from each search. The titles and abstracts of all identified articles were reviewed, and the included studies were further manually searched of their bibliographic references. Duplicate publications were removed after all databases and reference lists were searched. Whenever it was deemed necessary to finalize a decision about inclusion, the full article was reviewed.

Study inclusion criteria were:

(1) Studies that tested a group of healthy subjects.
(2) Trials in which different TMS-based VLs (ie, single/sequence of few pulses, rTMS) were given to various pain brain areas (eg, primary motor cortex [M1], primary sensory cortex [SI], prefrontal cortex [PFC], and parietal cortex [PAR]) in an online mode, ie, during continuous experimental pain (eg, capsaicin/electrical-induced hyperalgesia, tonic heat) or time-locked to a noxious stimulus (eg, laser).
(3) Studies reporting at least one outcome measure assessing pain measurement (eg, intensity, unpleasantness, and localization)

Exclusion criteria were:

(1) Studies designed to address populations with a specific pathology such as neurological (eg, stroke and Parkinson disease), metabolic (eg, diabetes), or musculoskeletal (eg, osteoarthritis) deficits.
(2) Studies in which TMS-based VLs were applied offline.
(3) Studies in which no inferential statistics were reported.
(4) Studies reported in conference proceedings or posters.

### 2.2. Data extraction

A data extraction form was developed by the authors and it included the following details: the type of subjects' and their age range; the number of each sex in each subject group; the study design including within- or between-group design, same-day or different-days performance of VL/sham tests, and randomized or set test order; the VL methodology including the location, intensity, and duration of the TMS; a description of the sham methodology and whether a control condition was included; details about the pain stimulus including the type/modality, location, and response mode (ie, outcome measure); and finally, the main key findings (Table 1).

**Table 1:**
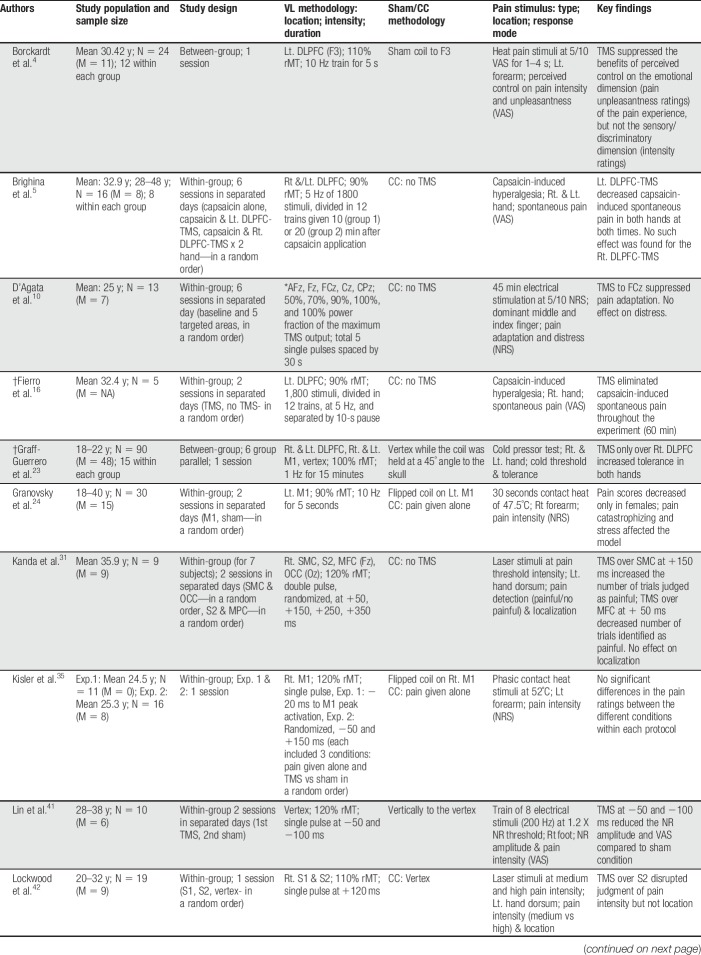

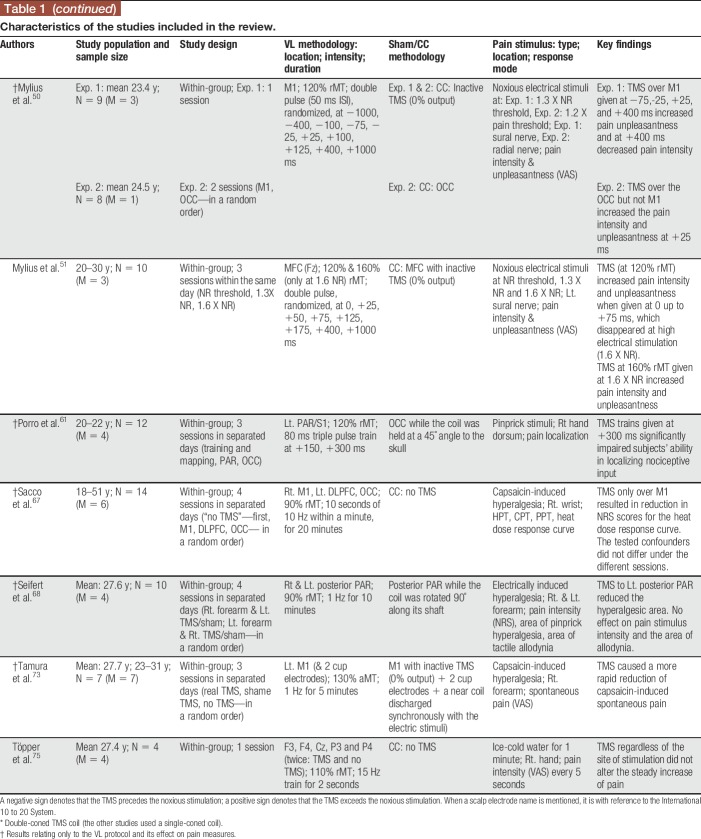
Characteristics of the studies included in the review.

## 3. Results

### 3.1. Study selection

**Figure 1.**
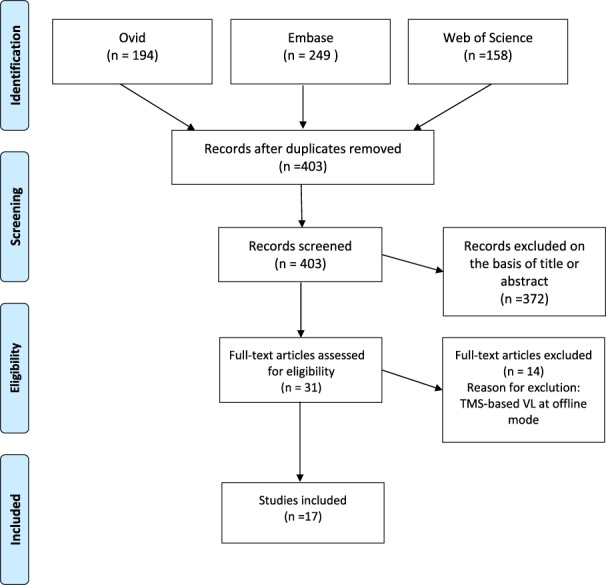
PRISMA flow diagram. This diagram depicts the flow of information through the different phases of this review.

The initial search of the 3 electronic databases resulted in a total of 403 studies, of which 31 were considered relevant on the basis of their title and abstract. Full-text review of these studies revealed that 14 of these studies performed an offline model of the TMS VL and were therefore excluded. Consequently, a final total of 17 studies were included in the review, detailed in Table 1. This selection process is summarized in Figure 1.

### 3.2. Subjects and study design

The 17 reviewed studies included 317 adults. Where the data were provided, the subjects were aged between 18 and 51 years with a mean age of 28 years. Two studies had a between-group design containing a total of 114 subjects (90,^23^ 24^4^); the remaining 15 reviewed studies used a within-group design. The number of subjects per group ranged from 4 to 30 subjects (a mean of 11.6 ± 5.7). However, only one study^35^ performed a power analysis calculation to justify the number of subjects they used in their study. Despite the study cohort including a mean of 50.1% males, the majority of the studies had a sex imbalance. Only 4 studies tested equal numbers of male and female subjects.^5,10,24,35^

In 10 of the within-group design studies,^5,10,16,24,31,41,61,67,68,73^ the VL interventions to different brain sites, including sham, were conducted in separate sessions on different days. Moreover, in 12^5,10,16,24,31,35,42,50,61,67,68,73^ of the within-subject design studies, the order of the stimulated areas was randomized or counterbalanced among participants.

Only 2 studies^24,67^ provided a control for potential confounders. Granovsky et al.^24^ included in their statistical model the following covariates: resting motor threshold (rMT), heat pain threshold, pain catastrophizing, stress, and fear of pain, due to their potential influence on pain perception and the TMS effect. They found that pain catastrophizing and stress affected the model. Sacco et al.^67^ tested whether positive and negative effects or pain catastrophizing account for the variability in pain reports, but did not find any such influence.

In the study by Sacco et al.,^67^ the investigators were blinded to the different TMS applications (ie, TMS or sham). Seifert et at.^68^ mentioned blinding but it is not clear whether the experimenters were blinded in addition to the participants. All the remaining studies did not report on blinding of the experimenters.

### 3.3. Virtual lesion intervention

The stimulated cortical areas were M1 in 6 studies^23,24,35,50,67,73^; S1 in 1 study^42^; sensory-motor cortex (SMC) in 1 study^31^; secondary somatosensory cortex (S2) in 2 studies^31,42^; medial frontal cortex (MFC) in 2 studies^31,51^; dorsolateral PFC (DLPFC) in 5 studies^4,5,16,23,67^; the vertex in 1 study^23,41^; and PAR in 2 studies.^61,68^ A further 2 studies used for their stimulation protocol the international 10-10 or 10-20 system of cortical electrode placement including frontocentroparietal electrodes^10,75^ (Ref. 10: AFz, Fz, FCz, Cz, CPz; Ref. 75: F3, F4, Cz, P3 and P4). Finally, one study^10^ used a double-coned coil to stimulate deeper structures, ie, the cingulate cortex.

The TMS coil location on brain areas other than M1 was based on (1) the individual brain anatomy using a high-resolution structural magnetic resonance imaging of the brain,^68^ yet not always in all participants^5,61^; (2) the location of scalp electrodes based on the International 10-10 or 10-20 system^4,10,31,51,75^; (3) the location in relation to M1, which was defined based on the motor-evoked potentials^16,23,42,67^; or (4) anatomical landmarks.^31,67^

Most studies used an intensity of between 90% and 120% of the rMT using the figure of 8 coil to elicit a VL. However, the intensity was adjusted relatively (130%) to the active MT in one study^73^ and with regard to the TMS output in another study.^10^

The TMS characteristics differed between studies and included single,^10,35,41,42^ double,^31,50,51^ or triple^61^ pulses time-locked to the pain stimulus onset or to the target brain area activity based on previous electroencephalography or magnetoencephalography studies. However, Kisler et al.^35^ was the first to time-lock the TMS to the brain area (M1) pain-related activity based on individual cortical-evoked potentials. In studies where rTMS was applied, the protocols included stimulation at either 1 Hz,^23,68,73^ 5 Hz,^5,16^ 10 Hz,^4,24,67^ or 15 Hz^75^ for 5 seconds^4,24^ or for a longer period from a few minutes up to 20 minutes^5,10,16,23,67,68,73^ during a long-lasting pain stimulus. An exception to this was a study by D'Agata et al.^10^ who applied single pulses at gradually increased intensities along a tonic stimulation. The following are the time windows that were used for TMS at different target brain sites (where a negative sign denotes that the TMS precedes the noxious stimulation and a positive sign denotes that the TMS follows the noxious stimulation): S1: +120 ms^42^; S2: +50 up to +350 ms^31,42^; SMC: +50 up to +350 ms^31^; M1: −1000 up to +1000 ms^35,50^; MFC: 0 up to +1000 ms^31,51^; vertex: −50 and −100 ms^41^; and PAR: +150 and +300 ms.^61^ In all the within-subject studies, the order of the different time windows for TMS were randomized, except in the studies by Porro et al.^61^ and Lin et al.,^41^ where randomization was not reported.

### 3.4. Control interventions

A sham procedure was used in 6 studies and included either a sham coil,^4,73^ a flipped coil,^24,35^ or a coil that was held at 45°^23,61^ or 90°^41,68^ to the skull. Moreover, Granovsky et al.^24^ and Kisler et al.^35^ added a control condition of pain stimulation given alone, in addition to a sham condition. In 2 studies, the TMS that was applied on untargeted brain areas such as the vertex or the OCC served as a control condition.^42,50^ Kanda et al.^31^ and Sacco et al.,^67^ although not stated as a control condition, used the TMS applied on the OCC as an independent variable in their analyses, among other areas of interest. In 2 studies, an inactive TMS (0% output) was applied on the targeted brain areas^50,51^ and in another 6 studies, no TMS served as a control condition.^5,10,16,31,67,75^

### 3.5. Pain stimulus characteristics

In the studies where the TMS was time-locked to the pain stimulus^31,41,42,50,51,61^ or to the targeted brain areas' pain-related activity,^35^ brief pain stimuli were used including laser,^31,42^ pinprick,^61^ electrical (×1.2, ×1.3, ×1.6 above the nociceptive reflex),^41,50,51^ and heat stimuli.^35^ When using rTMS, a prolonged pain stimulus was applied such as heat pain,^4,24^ cold pressor,^23^ ice-cold water,^75^ electrical,^10^ or capsaicin/electrically induced hyperalgesia.^5,16,67,68,73^ Painful stimulation was applied to sites on the upper limb, but when noxious electrical stimulation was applied, it was either to the upper limb^10,68^ or lower limb.^41,50,51^

### 3.6. Outcome measures

Assessments were conducted before intervention and during intervention in all studies. In 15 studies, at least one outcome measure examined pain intensity that was either rated or categorized (ie, painful vs nonpainful; medium vs high).^4,5,10,16,24,31,35,41,42,50,51,67,68,73,75^ Three of these studies included pain unpleasantness as an additional quantitative pain testing parameter.^4,50,51^ A further 3 studies tested pain localization abilities.^31,42,61^ Some studies applied pain threshold (heat, cold, and pressure)^23,67^ and tolerance^23^ tests. Finally, one study^41^ evaluated the nociceptive reflex amplitude.

No specific stimulated brain areas were targeted for the outcome measures evaluated in all the studies. When testing pain intensity judgment and ratings, the S1,^42^ S2,^42^ M1,^24,35,41,50,67,73,75^ MFC,^51^ cingulate,^10^ DLPFC,^4,5,16,67,75^ PAR,^68,75^ and vertex^41^ were the targeted brain areas. However, for changes in pain unpleasantness ratings, the M1,^50^ MFC,^51^ and DLPFC^4^ were exposed to TMS. To investigate certain targeted brain areas' roles in pain localization, VLs were applied to S1,^42^ S2,^31,42^ SMC,^31^ MFC,^31^ and the PAR.^61^ The M1, vertex, and the DLPFC^23,67^ were exposed to TMS to test their involvement in the processing of pain threshold and tolerance. The SMC, S2, and MFC^31^ were tested for their involvement in the judgment of pain (pain vs no pain) around the threshold intensity (pain threshold). Finally, one study^10^ applied VLs to the cingulate cortex to investigate its role in pain adaptation.

### 3.7. Virtual lesion effects

The VL effects described here are evaluated based on the pain processing functions that were tested. When testing the function of pain intensity judgment, it was found that single-pulse VL to S2, and not to S1, applied 120 ms after the pain stimulus^42^ disrupted the decision as to whether the pain intensity is high vs medium (no pain scores were obtained in this study). As for pain intensity coding, Tamura et al.^73^ induced VL to M1 and found a significant pain reduction compared with the sham condition, whereas 2 other studies^24,35^ reported no such effect vs a sham TMS. Sacco et at.^67^ and Mylius et al.^50^ also found pain reduction during VL to M1. In the latter study, the VL was compared with inactive TMS, whereas in the former, it was compared with VL applied to the OCC. The vertex involvement in pain ratings was evident when Lin et al.^41^ applied a VL before the pain stimulus initiation and found pain reduction compared with sham TMS. Yet, Töpper et al.^75^ did not find any change in pain ratings when a train of TMS was applied at Cz as compared to no TMS condition. When referring to the involvement of prefrontal brain areas in pain intensity encoding, a VL targeted to the MFC caused an increase in pain intensity vs inactive TMS.^51^ Reverse effects were found when TMS was applied to the DLPFC with either no effect^4,67,75^ or an elimination of spontaneous pain^5,16^ due to capsaicin-induced hyperalgesia. Moreover, 2 studies investigating the role of the PAR cortex in pain intensity coding^68,75^ found no change in pain ratings, whereas Sceifert et al.^68^ found a reduction in the area of hyperalgesia. Pain adaptation was investigated by applying a VL to the cingulate cortex^10^ but only a VL applied to the frontoventral area successfully suppressed pain adaptation.

Two studies by Mylius et al.^50,51^ investigated the role of M1 and MFC in pain unpleasantness ratings. They found that VL time-locked before (M1) and after (M1 and MFC) noxious stimuli resulted in an increase in pain-related unpleasantness ratings, whereas VL to the DLPFC suppressed the perceived control on the emotional dimension of pain.^4^ Furthermore, deep TMS targeted to induce a VL of the cingulate cortex revealed no effect on distress.^10^

Pain localization was investigated by applying VL to various brain areas. Virtual lesion to S1, S2, SMC, and MFC^31,42^ had no effect on pain localization when given after pain stimuli at threshold, medium, and high intensities. However, when applied to the PAR cortex, it impaired the ability to localize nociceptive stimuli.^61^

A variety of brain areas were stimulated to induce a lesion that may affect the pain threshold or pain detection. These included the S2,^31^ SMC,^31^ M1,^23,67^ MFC,^31^ DLPFC,^23,67^ and the vertex.^23^ Virtual lesion over the SMC and MFC after painful stimuli increased the number of trials reported as painful.^31^ Conversely, stimulating other brain areas did not yield any effect on the pain thresholds of heat, cold, pressure, or laser modalities.^23,31,67^ Finally, the pain tolerance measure was tested under VL induction on M1 and the DLPFC,^23^ and an increase in tolerance was found only when applied to the DLPFC.

**Figure 2.**
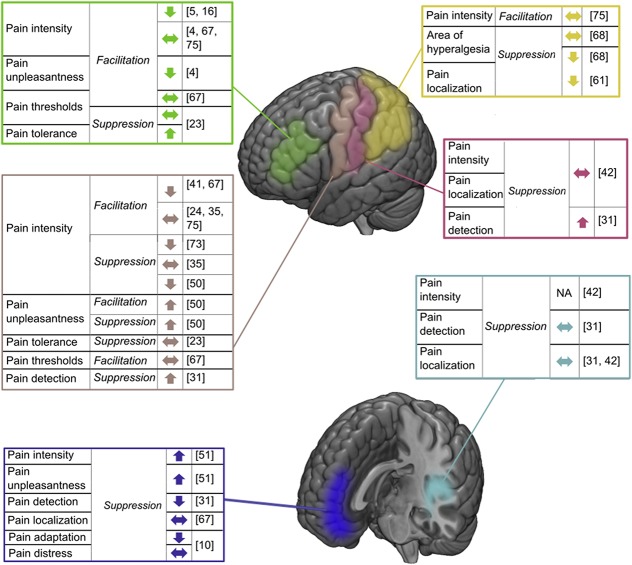
Graphical description of the VL effects on brain–behavioral relationship in pain. The targeted cortical brain areas are colored as follows: primary motor (M1; beige), primary somatosensory (S1; pink), secondary somatosensory (S2; pale blue), medial frontal (purple), dorsolateral (green), and parietal (yellow). For each cortical area, the pain tested behavior, the expected neurophysiological effect, ie, suppression or facilitatory, and the direction of the behavioral effects are presented. References are presented in brackets. VL, virtual lesion.

Figure 2 summarizes the expected neurophysiological effect and the direction of behavioral outcome of the VL effects on different brain areas. According to the literature, a suppressed neurophysiological effect is expected in response to ≤1 Hz rTMS or to single-pulse protocols given after the pain stimulus onset (due to the decreased signal-to-noise ratio of the neuronal activity). In line with this, a facilitatory neurophysiological effect is expected in response to >1 Hz (usually for the ≥5 Hz) rTMS or for the single pulses given before or at the onset of the pain stimuli. However, it is important to note that other stimulation parameters such as stimulus intensity may affect the neurophysiological response.

The following results are presented concerning various brain areas that were stimulated to test their involvement in different dimensions of pain processing. S1 and S2 were stimulated in a suppression mode and no effect was found on pain intensity ratings and localization.^31,42^ In addition, stimulation of S1 (also in a suppression mode) revealed an increased number of trials judged as painful, whereas no such effect was found for S2.^31^ M1 was stimulated either in a facilitation or a suppression mode; although no effects were found on pain threshold and tolerance,^23,67^ contradictory findings were identified on pain intensity^24,35,41,50,67,73,75^ and unpleasantness^50^ ratings with opposite behavioural effects to that expected neurophysiological effect or no effects at all. Parietal cortex stimulation in facilitation^75^ or suppression^68^ modes revealed no effect on pain intensity ratings. However, stimulation in a suppression mode reduced the area of hyperalgesia^68^ and significantly impaired localization abilities.^61^ The MFC was tested in a suppression mode of TMS while pain intensity and unpleasantness, as well as pain detection, adaptation, and localization functions were evaluated. Pain intensity and unpleasantness ratings increased^51^ and the number of trails identified as painful also increased,^31^ whereas adaptation^10^ decreased, with no effect on pain localization.^31^ Finally, DLPFC stimulation in a facilitation mode caused an inhibitory effect on pain intensity^5,16^ and unpleasantness^4^ ratings. Other studies found no effects on pain intensity^4,67,75^ and pain threshold.^23,67^ In addition, pain tolerance increased due to stimulation of the DLPFC in a suppression mode.^23^

## 4. Discussion

This is the first study to overview results of studies that tested the effect of TMS-induced virtual brain lesions on experimental pain characteristics. In this review, we included studies where the VLs were induced in healthy subjects through single pulse/seconds (this definition refers to the stimulation with single/double/triple or a sequence of 5 stimuli) TMS given at precise predetermined time points (ie, before, at the onset, or after the painful event, in terms of tens of milliseconds), or through rTMS trains delivered in the “online” mode (ie, simultaneously with tonic pain stimulation). According to the best of our knowledge, 17 studies with a total of 385 participants fitted these inclusion criteria and were included in this study. Overall, the studies tested the VL effect on various pain measures in response to radiant or contact heat, capsaicin intradermal injection or patch application, mechanical pricking, cold pressor, and noxious electrical stimuli. The VLs were directed to cortical areas associated with pain detection, coding, or localization such as the S1, S2, SMC, or PAR cortex, or to cortical structures activated by pain stimuli but also associated with antinociception such as the M1, MFC, and DLPFC, with or without control conditions. In our view, the main key finding of this review is that independently of the methodological characteristics, most of the described VL protocols efficiently affected psychophysical pain characteristics, although with a high variability of the modified pain responses (pain inhibition, pain facilitation, or no response).

### 4.1. Virtual lesion induced by single pulse/second transcranial magnetic stimulation

Eight studies used single (3 studies), double (3 studies), triple (1 study), or a sequence of 5 single pulses of increased intensity (1 study) for the VL stimulation protocols. The TMS pulses were delivered at different time points relative to the pain stimulus. When directed to the S2 cortex^31,42^ 50 to 350 ms after the pain stimulus onset (noxious laser), the VL disrupted the judgment of pain intensity but not the location of the pain stimuli in one study by Lockwood et al.^42^ but had no influence on pain characteristics in a study by Kanda et al.^31^ However, when TMS was directed to the S1/SMC, the VL increased the number of stimuli that were detected as painful^31^ without affecting pain intensity coding and localization.^42^ Beyond different control conditions (vertex stimulation^42^ vs no TMS^31^), observation of a behavioral VL effect on the S2 but not on the S1 by Lockwood et al.^42^ with reverse results from Kanda et al.^31^ may be related to their different outcome measures; coding of pain levels^42^ vs pain detection.^31^ Several previous studies have suggested that S2 codes pain intensity.^9,18,29^ Accordingly, the VL effect on S2 provides clear causal evidence for a functional role of this cortical structure in the ability to discriminate the intensity of a painful stimulus. However, this interpretation should be taken with precaution due to the very close approximation of S2 to the dorsolateral insular cortex, which can be determined as a nonspecific perceptual way-station rather than a specific pain center.^12^ Interestingly, the judgments of pain intensity were significantly disrupted not only when comparing S2 with a control site (vertex), but also in comparison with TMS applied to S1.^42^ This finding points to distinct roles for S1 and S2 in pain perception despite their coactivation by nociceptive stimuli.^17,40,58^ For example, there is evidence from behavioral and electrophysiological studies that S1 cortical nociceptive neurons encode various sensory features of pain^6,32,33^ including pain localization^30,37^ due to their relatively small receptive fields.^32^ Thus, under certain stimulation conditions, S1-directed VL can disrupt pain localization, whereas the S2-directed VL can disrupt pain intensity. Another possible explanation for this differential responsiveness of S1 and S2 to VL manipulations is the higher reproducibility of the S2 vs S1 activation by noxious stimulation.^74^

Among the 3 studies that applied VL to the SMC, S1, or PAR cortex,^31,42,61^ only Porro et al.^61^ found an impaired ability to locate pain. This may be due to Porro et al.'s distinct methodology. Namely, they used mechanical pricking stimuli that activate both A-beta and A-delta fibers, along with longer (triple vs single or double stimuli) and later (+300 vs +150 ms) application of the TMS. The VL effect on the PAR cortex may be also attributed to the involvement of this cortical region in spatial discrimination of noxious stimuli,^54^ attention to pain,^59,76^ and somatosensory integration.^44^

Although the effect of single TMS pulse VL on cortical areas associated with the encoding of pain intensity generally resulted in a disruption of pain coding and localization, the VL protocols directed to brain areas associated with inhibitory pain modulation brought more mixed results. Double-pulse TMS directed to the MFC (and defined by the authors as anatomically associated with the anterior cingulate cortex [ACC]) at 50 ms after the noxious laser stimuli resulted in an antinociceptive effect with fewer trials reported as painful.^31^ Conversely, in another MFC-directed VL protocol (+75 ms, electrical pain stimuli), pain and unpleasantness scores increased.^51^ Furthermore, anterior midline TMS stimulation using a double-coned coil orientated to the ACC and comprising 5 stimuli given at increasing intensities resulted in decreased adaptation to noxious electrical stimuli.^10^ These contradictory findings from 2 MFC/ACC-directed VL protocols may be attributed to the various functions of the ACC in pain. The ACC has a role in pain perception because its activation is often detected in various pain-stimulation protocols^2,60,64^; consequently, the expected inhibitory effect of VL would result in a decrease in pain. However, because the ACC also takes an important role in initiating and mediating endogenous analgesia,^11,52,57^ VL could also result in an increase in pain.

Three of 8 single-pulse VL protocols described in this review were directed at the M1.^35,41,50^ Although not fully understood, many studies point to a relation between pain and motor cortex activity.^45^ Similar to the ACC, increased M1 activation has been reported in many imaging and electrophysiological studies on experimental pain,^19,22,35^ with the main expected effect of its activation to be a pain decrease.^21,46^ Therefore, considering the analgesic effect of M1 activation on experimental pain, which is associated with increased M1 excitability,^26,63^ using single pulse/second VL protocols where TMS is given after the pain onset, the expected neurophysiological effect would be suppression of M1 activity along with a pain increase. In case of the application of single TMS stimulus before the pain stimulus, the opposite occurs, namely a facilitating neurophysiological effect along with pain attenuation. The studies presented in this review show findings in both directions: the expected change in pain perception vs the opposite to what would be expected. Double or single TMS pulses directed to the M1 at −100 to +25 ms relative to the pain stimulus onset increased electrical pain unpleasantness in one study,^50^ but decreased pain ratings in another study.^41^ Opposite to what would be expected, decreased pain intensity was also induced by later (+400 ms) single-pulse TMS.^50^ Conversely, 2 other studies applying single-pulse TMS-induced VL of the M1 did not reveal any changes in pain characteristics. More specifically, the effect of single-pulse TMS was no different from a sham TMS when the pulses were synchronized with the electroencephalography-related individual peak of the pain-evoked M1 activity (−20 ms), or given at −50 to +150 ms relative to the phasic contact heat stimuli.^35^ Thus, there is a discrepancy in the findings, with some reports showing a change in pain due to VL, whereas others observe no such effects. This might be related to various methodological factors, as will be discussed below.

### 4.2. Virtual lesion induced by repetitive transcranial magnetic stimulation

The rTMS stimulation protocols varied widely in their applied stimulation frequency (between 1 and 15 Hz) and duration (between 5 seconds and 20 minutes). All the studies applied rTMS on cortical areas associated with exerting antinociception (ie, the prefrontal cortices and M1), with the PAR cortex as an additional site of stimulation.^68,75^ There was one study that was an exception and instead directed TMS to solely the parietal cortex.^68^ Two of the reviewed studies reported no effect on pain intensity after a 1-Hz stimulation for 10 minutes applied during electrically induced pain^68^ or after a 15-Hz stimulation during ice-cold water hand immersion^75^; however, the former observed a significantly reduced area of hyperalgesia,^68^ with no behavioral pain effect for the 15-Hz stimulation at any stimulated site tested in this study.^75^ Both studies on the PAR-directed stimulation had no effect on pain intensity. Although inconsistent, these findings confirm the role of the PAR cortex in spatial discrimination of noxious stimuli rather than in pain coding.

Six studies directed trains of rTMS to the PFC/MFC including the DLPFC. The DLPFC is a large and functionally heterogeneous brain region implicated in many complex cognitive processes such as appraisal of pain, cognitive and emotional control, as well as top down modulation of pain.^69^ One study used a 1-Hz stimulation frequency to the right DLPFC and reported increased tolerance to a cold pressor test.^23^ This finding is surprising because it has been shown that low-frequency stimulation (<1 Hz) reduces neuronal excitability^8,48,66^; therefore, the expected inhibitory effect from a 1-Hz stimulation directed to a cortical area associated with antinociception would be a pain increase. A possible explanation for this unexpected finding would be an indirect stimulation effect of the DLPFC on other structures associated with antinociception through transynaptic connections.^34,72^ Indeed, TMS affects adjacent brain areas by the physical spreading of the induced neuronal electrical activity, and also affects long-distant brain areas by the propagation of action potentials along corticocortical and corticosubcortical connections.^78^ Therefore, rather than locate a given function to a specific brain structure, the functional connectivity between brain structures may mean that a transient disruption of a given cortical region may also tell us about the capacity of the rest of the brain to adjust to it (ie, react or adapt). This notion is supported by recent reports about the positive relationship between the DLPFC–subgenual ACC functional connectivity, and the clinical efficacy of rTMS treatment for depression.^20^ Across 5 studies that applied facilitatory rTMS (at 5–15 Hz) on the DLPFC, 2 studies did not find a significant effect on pain modulation induced by capsaicin cream application or in the pain perception from noxious heat stimuli given on the hyperalgesic skin^67^ or an ice-cold water hand immersion,^75^ which indeed may be a cumulative result from such a remote effect of neuromodulation. This is in contrast to the results of the 3 remaining studies that did show reduced pain ratings to capsaicin-induced pain^5,16^ and reduced unpleasantness ratings to trains of noxious heat stimuli^4^ in response to left DLPFC stimulation. These 3 studies therefore confirm the functional role of the DLPFC in pain inhibition.

Five studies used rTMS to test the VL effect on M1. Two of them applied inhibitory (1 Hz) stimulation with contradictory results; there was no effect when rTMS was applied during a cold pressor test in one study,^23^ whereas another study reported a speeding up of the gradual reduction of acute pain from an intradermal capsaicin injection.^73^ According to the authors, the latter finding may be related to the simultaneously modified activity of the ACC and prefrontal cortices due to distant corticocortical connection, as described above. This possible widespread effect could also be attributed to the higher stimulation intensity used in the latter study: 130% of the rMT^73^ vs stimulation at the rMT level.^23^

Among the 3 studies that used high-frequency rTMS, one reported no effect on ice-cold water pain^75^ or noxious tonic contact heat,^24^ wherase a significant analgesic effect was found for 10-Hz rTMS applied to noxious heat stimuli given on hyperalgesic skin^67^. The latest findings are in line with the results of many clinical studies pointing to M1 as an important stimulation target in chronic and neuropathic pain treatments.^28,38,39,47,53,65^ The stimulation of M1 induces a cascade of synaptic events, which modulates activity in the brain structures of the pain neuromatrix and potentiates the activity of the brain's antinociceptive system.^21,43,46^ Interestingly, despite no significant overall effect, the 10-Hz rTMS VL induced a reduction in heat pain in females.^24^ Among the possible contributing factors related to this sex-related effect is the higher transcallosal inhibition of the TMS-evoked muscle responses in females,^13^ which is under the influence of gonadal hormones.^25^

### 4.3. A summary of the contribution of virtual lesion to our understanding of the role of different brain areas in pain processing

A VL was not efficient in disrupting the main functions that are related to S1, ie, pain intensity coding and localization. Although not clearly related to S2 function, these pain dimensions were also not disrupted when a VL was targeted to S2. Although the M1-directed VL did not affect pain thresholds and tolerance measures, it did affect the pain intensity and unpleasantness. Therefore, these bidirectional effects eliminate conclusive results. The PAR cortex stimulation successfully inhibited spatial dimensions of pain processing, had no effect on pain intensity rating, and usefully decreased the area of hyperalgesia. Virtual lesion applied to the MFC was expected to inhibit pain intensity and unpleasantness due to its role in pain modulation and emotional processing of pain, yet failed to do so. A VL on the DLPFC, a brain area that is involved in cognitive processing, successfully increased pain tolerance, which is known to be affected by motivational and cognitive processes. At the same time, contradictory results were found on pain thresholds, intensity, and unpleasantness measures. The partial success in disrupting the functions attributed to various pain processing brain areas point to the complexity of this procedure, and to methodological limitations that may contribute to the inconclusive results.

### 4.4. Limitations and methodological considerations

Despite an overall convincing picture of efficient induction of VL in pain-related brain structures, which subsequently modulated pain behavior, there are several important factors that can interfere with the conclusions. The first factor relates to the small number of subjects in some of the studies: a total of 4 to 30 participants for the within-subjects design, and 9 to 90 (divided into 6 groups) participants for the between-subjects design. None of the studies except one^35^ justified their sample size with a power analysis calculation. In our opinion, making conclusions on intervention efficacy from such unjustified small samples may theoretically be a source for biased assumptions regarding the contribution of a given cortical region to pain processing. This is further compounded by the fact that the study groups were unbalanced in terms of sex; some study groups were comprised with males only^31,73^ or no males at all.^35^ Because there are reported sex differences in the performance of behavioral tasks in response to TMS or therapeutic interventions,^27,36,77^ including VL protocols,^24,36^ the sex-matched study population is an important condition to achieve reliable and valuable results of the VL effect. Furthermore, the high variability in study designs: within- or between-subjects comparisons; experimental sessions on separate days or the same day; the inclusion or not of a control condition or sham stimulation, and particularly critical, the TMS type, and targeted brain locations, should all be taken into consideration. The analysis of studies presented in this review indicated that 6 of 17 studies did not use any control condition, yet 5 of them concluded that there was a VL effect. Proper sham stimulation and stimulation blinding (at least for the subject) are critical in neuroscience research to minimize the placebo effect on outcome measures. Placebo effects are inherent to every treatment or intervention. Multiply neurochemical and neuropharmacological mechanisms, along with cognitive and psychosocial factors may change the circuitry of the brain's modulatory networks contributing to a placebo effect.^3,7^ Moreover, there is an increasing number of reports observing that drugs used for years in pain clinics fail to provide a treatment efficacy greater than placebo.^14,15,62^ Therefore, we strongly opine that a placebo condition by means of a sham TMS or stimulation of nonactive remote brain areas is mandatory for VL-induction pain studies.

To conclude, most studies on TMS given in the online mode to induce the local transient “VL” in various structures of the pain neuromatrix were accompanied by a behavioral effect (ie, a decrease or increase of pain responses). However, in some studies, the VL had no effect. This can be explained based on the fact that the pain perception process is complex and consists of a large network of brain areas that are structurally and functionally interconnected. Accordingly, VL to one brain area may not be powerful enough to induce an observable effect on the tested pain behavior. Possibly, more intense interventions such as simultaneous stimulation of multiple brain areas that compose the pain neuromatrix may achieve such an observable and convincing effect.

Similar to results from neurocognitive studies, mixed findings on the relationships between neural activity and pain perception may be associated with stimulation parameters and testing conditions. These encompass the baseline state of the stimulated brain, the stimulation intensity and frequency (for the rTMS-VL), and the ability to successfully take into account factors such as sample size, sex balance, and control conditions. An additional idea for future studies would be to use e-field modeling approaches to improve the spatial accuracy together with per-pulse dose approximation for magnetic stimulation. We also advise that it should be an obligatory standard of all TMS research to report all details of the TMS protocol because methodological factors influence the magnitude and direction of the TMS-induced behavioral effects. Today, the main cortical sites for the rTMS treatment of pain are restricted to the M1 or DLPFC. The “VL” approach will facilitate our understanding of the functional relevance of other cortical targets for pain processing, and thus justify future neuromodulation treatment approaches.

## Disclosures

The authors have no conflict of interest to declare.
